# Processing of PVDF/Nb^5+^ and Sr^2+^-Doped PZT Composites for Applications in Advanced Dielectric Insulation: Effect of Ceramic Loading on Structure, Mechanical Properties, Electrical Properties, and Thermal Aging Behavior

**DOI:** 10.3390/polym18192417

**Published:** 2026-10-03

**Authors:** Alina Ruxandra Caramitu, Romeo Cristian Ciobanu, Alina Iulia Dumitru, Eduard-Marius Lungulescu, Magdalena Valentina Lungu, Virgil Emanuel Marinescu, Alexandru Chirica

**Affiliations:** 1 National Institute for Research and Development in Electrical Engineering (ICPE-CA), Unirii no. 3131, District 3, 030138 Bucharest, Romania; alina.dumitru@icpe-ca.ro (A.I.D.); marius.lungulescu@icpe-ca.ro (E.-M.L.); magdalena.lungu@icpe-ca.ro (M.V.L.); virgil.marinescu@icpe-ca.ro (V.E.M.); 2 Department of Electrical Measurements and Materials, Gheorghe Asachi Technical University, Bdul. D. Mangeron 71, 700050 Iasi, Romania; alexandru.chirica@student.tuiasi.ro; 3 All Green SRL, Str. I. Bacalu no. 5, 700023 Iasi, Romania

**Keywords:** PVDF, PZT, Pb(Zr_0.52_Ti_0.48_)O_3_ doped with Nb^5+^ and Sr^2+^

## Abstract

In this study, four types of materials suitable for use as special insulation were produced. Polyvinylidene fluoride (PVDF) served as the polymer matrix, while PZT powders (a Pb(Zr_0.52_Ti_0.48_)O_3_ system doped with Nb_5+_ and Sr_2+_) were used as the filler material; the method for producing these powders is also described. The mass concentrations, expressed as PVDF/PZT ratios, were 100/0, 95/5, 90/10, and 80/20. Characterization analyses and tests were conducted to verify the materials’ suitability as insulators. Specifically, the materials underwent structural (SEM and FTIR), chemical (EDS), mechanical, and dielectric testing (measuring tan δ, ε_r_, ρ, and E_str_). Additionally, resistance to degradation at the maximum operating temperature was evaluated through thermal resistance tests at 150 °C. Finally, the remaining service life prior to complete material degradation—and the consequent need for replacement—was determined. All characterization tests indicated that the composite material with a 5% PZT powder concentration—which exhibits the best structural, mechanical, dielectric, and thermal properties—represents the optimal composite variant for insulation applications. Potential applications for these materials include coatings for electric field grading and voltage control, as well as insulation for high-voltage transients and impulses.

## 1. Introduction

In recent years, polymer composite materials [1] have attracted significant interest in the fields of sensors [2], actuators [3] and energy harvesting [4]. The study of the behavior of polymeric composites has attracted significant attention due to their wide range of industrial applications, especially in the aerospace [5], aeronautics [6,7] and automotive engineering, industries, where they are used as sensors and actuators [5].

Polymer matrix composites exhibit temperature-, frequency- and time-dependent properties [8,9,10,11]. A good dielectric material should have a high dielectric constant (ε_r_), an ultra-low loss tangent (tanδ), a high electrical breakdown resistance (Eb), and should achieve a high discharge energy density (Udis) and a high discharge efficiency (η) [12,13].

The most common polymer with a high dielectric constant is PVDF. It has been studied by the authors of [14,15] and is considered to be the best alternative to classical piezoelectric materials [16,17]. PVDF maintains its performance in a wide range of demanding environments, which makes it a versatile material for various advanced applications [16].

Many researchers have tried to introduce nanoparticles, such as graphene and carbon nanotubes (CNTs), into the PVDF matrix [18]; others have introduced PZT and BT into its matrix to increase its rigidity [19,20,21]. Given that three-phase polymer composites are promising materials for the fabrication of electronic device components—and considering that the qualitative and quantitative composition of these composites significantly influences their functional properties, particularly their dielectric characteristics—researchers [22] sought to optimize ε_r_ and tan δ by using Ag-coated K_2_Ni_0.93_Ti_7.07_O_16_ (KNTO) ceramic material embedded in a PVDF polymer matrix at volume concentrations of 7.5%, 15%, 22.5%, and 30%; they concluded that KNTO@Ag(1 wt%)-PVDF composites can serve as dielectric materials for passive components in flexible electronics.

Some researchers [23] have demonstrated that the incorporation of 20% PZT significantly improves the tensile properties of the composite, increasing its suitability for actuation purposes.

The iso-field micromechanical model developed in [24] was used to estimate the effective properties of composites. In addition, the effects of phase properties, orientation angles, volume fractions and particle shape on the effective properties of composites were extensively analyzed in [25,26], which introduced a new modeling approach to predict the electromechanical properties of composites. Some researchers [8,27] studied the viscoelectroelastic behavior of two-phase composite materials, in which lossless particles are embedded in a lossy matrix, to include the effects of particle coating on the properties of a viscoelectroelastic composite.

A wide range of polymers and ceramic materials have been used to obtain polymer–ceramic composites [28]. Of the ceramic materials used, those with dielectric/piezoelectric/ferroelectric properties are of great interest. Among these, we can list materials such as barium titanate (BT), lead zirconate titanate (PZT), lead magnesium niobate–lead titanate (PMN-PT), etc. [28,29].

In our work, we obtained three recipes for polymer composite materials with applications in the realization of special insulation. The polymer matrix used was PVDF, which has a dielectric constant at 100 Hz of 7.5478. The fillers were PZT powders (a Pb(Zr_0.52_Ti_0.48_)O_3_ system doped with Nb^5+^ and Sr^2+^). Doping the ceramic PZT phase influences its crystal lattice dynamics, which directly affects its interfacial compatibility, local field distribution, and dipole alignment when integrated into the polymer matrix. Introducing Niobium5+ and Strontium2+ co-doped PZT into a PVDF matrix turns standard lead zirconate titanate into a high-performance composite. For the PZT system, we used Nb^5+^ as a dopant for Ti^4+^/Zr^4+^ to reduce the grain size during the sintering process of the powder. We also used Sr^2+^ as a dopant for Pb^2+^ to improve the piezoelectric properties [29]. The softened lattice dynamics in Nb/Sr-PZT reduce the mechanical mismatch between the hard ceramic particles and the soft polymer matrix during stretching or thermal cycling, preventing micro-void formation and minimizing local dielectric field concentrations.

The mass concentrations of the obtained materials expressed by the PVDF/PZT ratio were 100/0, 95/5, 90/10 and 80/20. The materials were obtained by melt injection, thermally conditioned and characterized from a structural (SEM and FTIR analyses), chemical (EDS), mechanical, thermal and dielectric point of view. The criterion for evaluating the moment of the onset of degradation was the variation in the dielectric loss angle tangent. Dielectric tests were performed after each aging cycle. The beginning of degradation is evidenced by the sudden increase in the dielectric loss angle tangent and implied by the decrease in electrical resistivity to 30% of the initial value.

Following the SEM+EDS structural characterization tests, it was observed that the PZT powder is relatively uniformly distributed in the PVDF polymer mass. Mechanical tests confirm that, at 5% by weight of PZT powder, the interfacial adhesion between the filler and the polymer matrix is optimized, increasing the rigidity and strength by restricting the mobility of the polymer chain. In turn, the dielectric and temperature resistance tests qualify the composite material with 5% PZT as the material with the best insulating characteristics. Thus, following the characterization of these materials, the composite coded with 5% PZT was chosen as the optimal variant as special insulation for various electronic equipment.

The novelty of our approach is that it obtains PVDF/doped PZT composite materials by the classical method of processing plastic materials. According to our knowledge, the simultaneous integration of Nb^5+^ and Sr^2+^ into PZT and incorporation within PVDF matrix has not been studied by other researchers, according to the current literature.

The potential targeted applications of such composites would be as follows: (1) Electric Field Grading and Stress Control Coatings—used in high-voltage cable terminations, bushings, and switchgear to even out electric field distributions, reduce localized electric stress peaks, and prevent issues like partial discharge or surface flashovers. (2) High-Voltage Transient and Pulse Insulation—to be integrated into specialized pulsed-power systems and high-frequency transformer insulation layers, requiring high spatial capacitance combined with strong voltage tolerance. Some required properties for these applications include the following: (1) High dielectric permittivity, as PZT ceramic fillers are used to significantly enhance the composite permittivity beyond that of PVDF, enabling a high capacity for charge storage). (2) High dielectric breakdown strength, because the materials must endure intense electric field stress without failing electrically. Maintaining low field concentrations at filler–matrix interfaces helps achieve high breakdown levels of around 100 kV/mm. (3) Low dielectric loss to reduce internal heat generation and avoid thermal runaway during high-frequency or high-voltage AC operations (aiming for a tan delta less than 0.05). (4) High volume resistivity and low leakage current, as a high electrical insulation resistance is essential to prevent DC leakage current and minimize active power dissipation. (5) Interfacial compatibility and homogeneous dispersion, i.e., the PZT particles should be evenly distributed within the PVDF matrix, avoiding agglomeration or micro-voids that could lead to localized charge accumulation. (6) Thermal stability, i.e., the materials must retain high thermal stability up to its operational limits (typically between 120 and 150 °C), while maintaining optimal dielectric performance.

## 2. Materials and Methods

### 2.1. Materials

To obtain PVDF/PZT composite materials, the following raw materials were used:

- 3M™ Dyneon™ Fluoroplastic PVDF 6008/0001 (commercial powder) from 3M Science Applied (St. Paul, MI, USA) to life [30].
- PZT powder (powder obtained in our laboratory)—a Pb(Zr_0.52_Ti_0.48_)O_3_ system doped with Nb^5+^ and Sr^2+^ obtained by solid-state reactions. This system presented ε_r_ = 1180 and k_p_ = 0.42 [31].

### 2.2. Equipment and Methods

#### 2.2.1. Obtaining and Characterizing Doped PZT Powder

**Figure 1:**
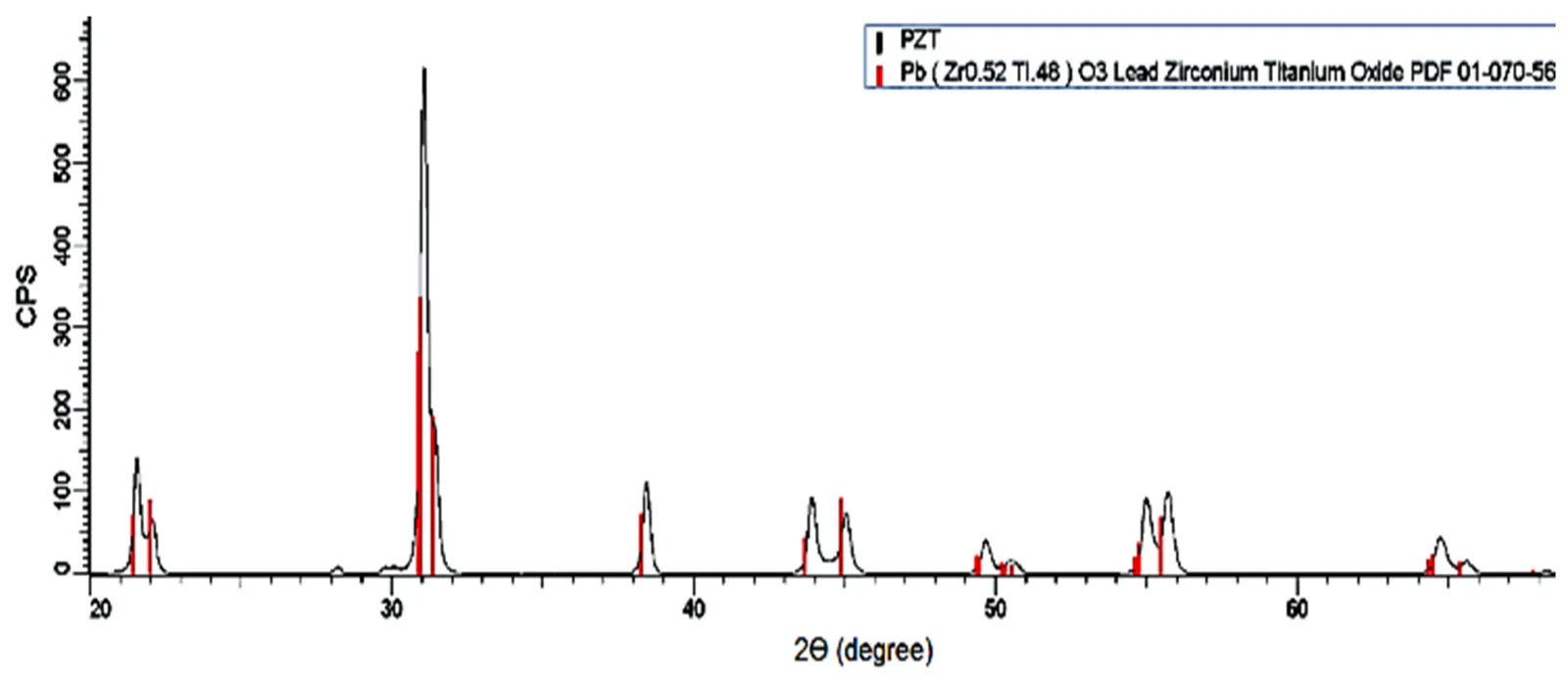
XRD pattern of PZT doped with Nb^5+^ and Sr^2+^, sintered at 1140 °C for 2 h.

The doped PZT powder was obtained by solid-state reactions, starting with high-purity raw materials. Thus, we used as raw materials the oxides PbO and Nb_2_O_5_ (Honeywell Fluka, Seelze, Germany), ZrO_2_ (Merck Group, Darmstadt, Germany), TiO_2_ (Sigma-Aldrich, Burlington, MA, USA), and carbonates SrCO_3_ (Carlo Erba, Cornaredo MI, Italy). The detailed method used to obtain the PZT powder doped with Nb^5+^ and Sr^2+^ is presented in the paper [32]. In order to embed the doped PZT powder in PVDF, after sintering at 1140 °C for 2 h, the powder was milled for 5 h using an Attritor mill (Union Process, Akron, OH, USA). The milling medium used was H_2_O. The identification of the phases formed after the sintering treatment was performed by X-ray diffraction (XRD). Figure 1 shows the X-ray diffraction spectrum obtained for the doped PZT composition [31]. The sintered composition presents the formation of a single phase with a tetragonal perovskite structure.

The dielectric properties (ε_r_ and tan δ) together with the piezoelectrical properties (electromechanical coupling factor (k_p_) and direct charge coefficient (d_33_)) are presented in Table 1 [32].

#### 2.2.2. Composites Manufacturing

The raw materials were dried in a Memmert oven (Memmert, Schwabach, Germany) for 2 h at 60 °C, then mechanically mixed in a Turbula-type mixer for 2 h. The composite materials were produced via melt injection using a Dr. Boy (Germany) injection molding machine with the following main characteristics: ϕ screw 28 mm, L/D ratio 18.6, calculated injection capacity 58.5 cm^3^, maximum material pressure 2200 bar, real injection capacity is 53 cm^3^. The mold in which all the samples were made presents in a single injection operation: 2 bar-type specimens for mechanical tests, 1 disk of 28 mm in diameter for dielectric tests and 1 disk of 12.9 mm for, structural tests, etc.

**Figure 2:**
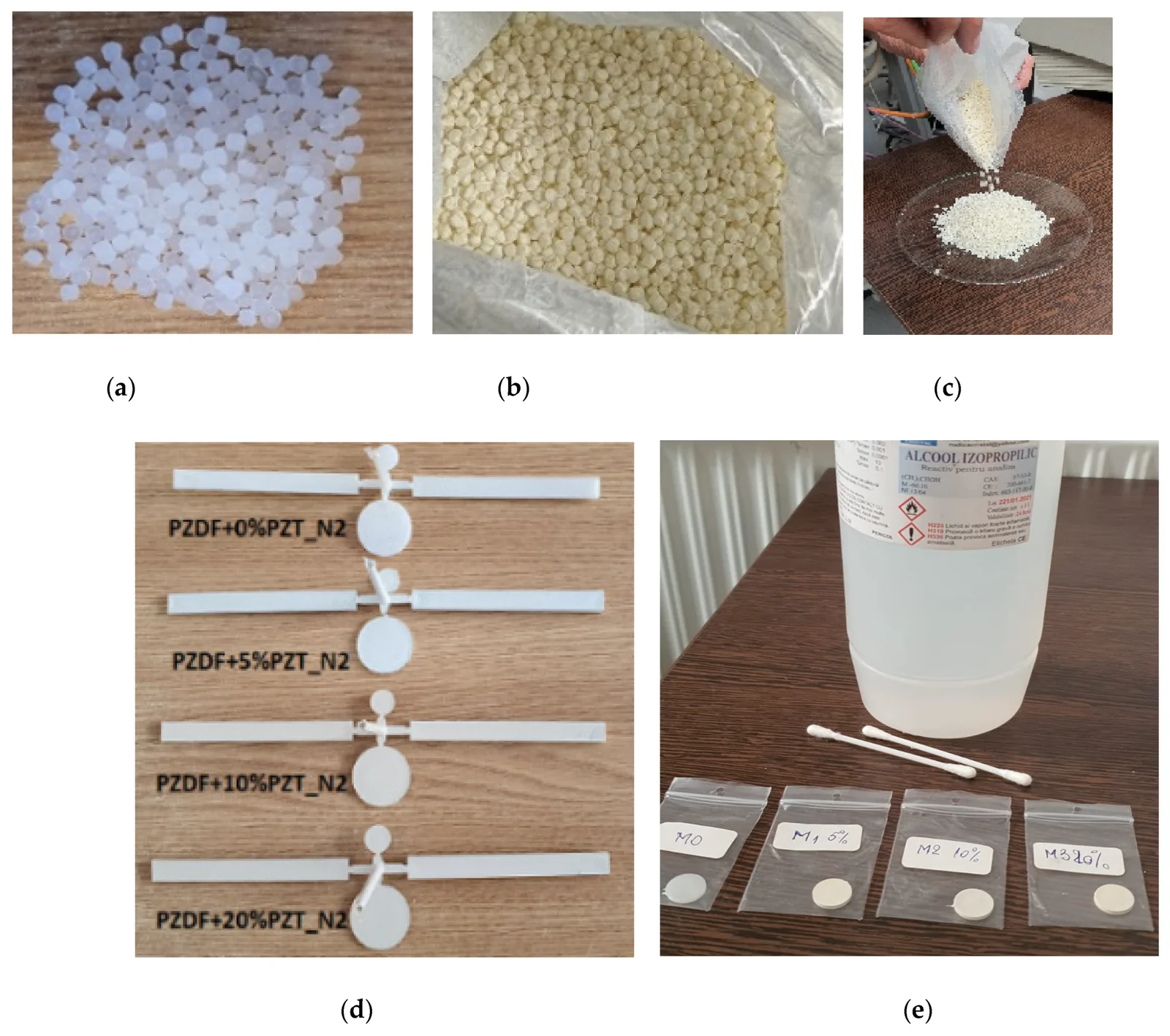
(**a**) PVDF granules; (**b**,**c**) deposition of PZT powder on the PVDF surface; (**d**) samples resulting from the melt injection process; (**e**) samples cleaned with isopropyl alcohol and dried at 70 °C for 15 min.

The processing temperature range on the 5 heating zones of the machine was 210–250 °C, with mold closing force = 120 kN and pressure = 110 bar. Figure 2 shows the deposition of PZT powder on the surface of the polymer granules, as well as the composite materials obtained.

Table 2 presents the codings and mass concentrations of the raw materials from which the composites were made.

**Figure 3:**
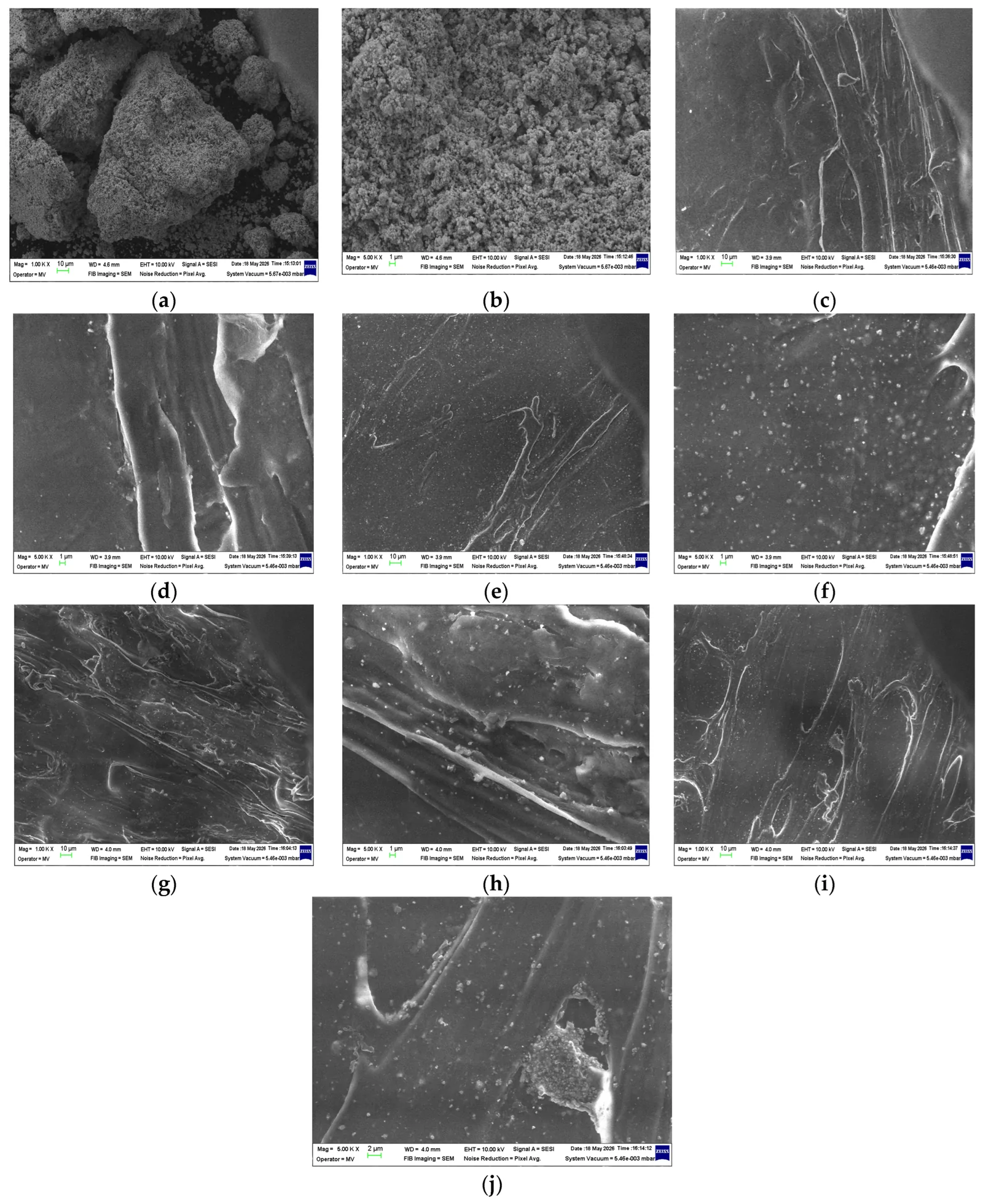
(**a**,**b**) PZT_N2 1000×, 5000×, (**c**,**d**) M0 1000×, 5000×, (**e**,**f**) M1 1000×, 5000×, (**g**,**h**) M2 1000×, 5000×, and (**i**,**j**) M3 1000×, 5000×.

Figure 3 shows the interface of the injection machine when obtaining the 3 concentrations of PVDF/PZT composite materials.

The temperatures of the injection machine screws in the 5 heating zones during the technological process of obtaining PVDF/PZT composites are presented in Table 3.

#### 2.2.3. Characterization Methods

##### SEM and EDS Analyses

SEM and EDS analysis of PZT powder as well as M0-M3 materials was performed using an Auriga field emission scanning electron microscope (FESEM) (Carl Zeiss, Oberkochen, Germany) equipped with a Canion focused ion beam (FIB) column (Orsay Physics, Fuveau, France) and an X-MaxN Energy-Dispersive Spectroscopy (EDS) Silicon Drift Detector (SDD) sensor with Aztec 3.0 software (Oxford Instruments plc, Abingdon, UK). SEM images were recorded at a 5 kV accelerating voltage. For sample uncoating, we employed CC local charge compensation with injected nitrogen in a sample chamber at 1000× and 5000× magnification, taking images of fractured samples. During the EDS analysis, we employed a 10 kV accelerating voltage and WD of 5 mm, and the EDS probe used was an Oxford Instrument X-MAX with a window measuring 50 square millimeters and Aztec-type acquisition software installed. For both SEM and coupled SEM-EDS analysis, we employed the Everhart-Thornley secondary electron (SE) detector with a Faraday cup situated in the sample chamber.

SEM (Scanning Electron Microscopy) analysis provided important information about the morphology, distribution and interface of the composite, while EDS (energy-dispersive x-ray spectroscopy) analysis provided information about the qualitative and quantitative elemental composition.

##### ATR/FTIR Spectroscopy

The structural changes induced by PZT incorporation and ageing were also investigated by ATR-FTIR spectroscopy. Pristine PVDF and PVDF/PZT composites containing 5, 10 and 20 wt.% PZT were analyzed both in the initial state and after thermal aging at 150 °C for 120 h. Spectra were collected using a JASCO FT/IR-4200 infrared spectrometer (JASCO, Tokyo, Japan) in the 4000–400 cm^−1^ spectral range, with a resolution of 4 cm^−1^ and 100 accumulations per spectrum. The spectra presented in this work correspond to the average of five spectra recorded at different positions on the sample surface, in order to minimize the influence of local heterogeneities and sample preparation-related surface defects. To allow a reliable comparison between samples, the spectra were baseline-corrected and normalized using the band at approximately 1400 cm^−1^, assigned to CH_2_ bending vibrations and used as an internal reference band [33]. Particular attention was paid to the 400–1600 cm^−1^ region, which contains the main phase-sensitive bands of PVDF, including the α-phase bands around 763, 795 and 975 cm^−1^ and the electroactive β/γ-related bands around 840 and 1275–1280 cm^−1^. The intensity ratios A_840_/A_763_ and A_1275_/A_763_ were used as semi-quantitative descriptors of the relative electroactive contribution and chain conformational ordering.

##### Mechanical Properties

The mechanical properties of the disk-shaped samples were evaluated via load-controlled instrumented indentation testing using a Micro-Combi Tester (MCT^2^) equipped with a diamond Berkovich indenter (CSM Instruments, Peseux, Switzerland). Nanoindentation measurements were carried out in accordance with the [34]. A Poisson’s ratio of 0.40 was assumed for all polymer-based samples. Indentation hardness (HIT), Vickers hardness HV, and elastic modulus (EIT) were determined using the Oliver and Pharr method [35]. For each sample, five indentations were performed, and the results are presented as mean values ± standard deviation (SD).

##### Dielectric Properties

Dielectric characterization evaluates the dielectric properties (ε, tan δ, and impedance) over varying frequencies and temperatures. These measurements are essential for evaluating the electrical behavior of materials and determining their applicability in various applications. Typical methods for dielectric characterization include impedance spectroscopy [36], dielectric relaxation spectroscopy [37], and broadband dielectric spectroscopy [38]. The dielectric properties, ε_r_ and tan δ, provide valuable information about the dipole’s interaction with electric fields, influencing its ability to store and dissipate energy [39].

The random nature of ε_r_ in the frequency range from 1 Hz to 1 kHz encourages researchers to consider data starting at 1 kHz [40]. In the low-frequency range, ε_r_ of PVDF exhibits fluctuations caused by interfacial polarization (Maxwell–Wagner–Sillars) [41] and space charge effects [42]. The tan δ of composites is a key parameter that measures the energy loss during electrical or mechanical oscillations. A lower tan δ (typically <0.1) signifies higher energy efficiency and reduced heat generation, making it advantageous for various applications [43,44].

In this work, the dielectric properties of the M0-M3 coded samples were determined by dielectric spectroscopy using a Solartron 1260A dielectric spectrometer (Solartron Analytical, Farnborough, UK). The measurements were recorded using an electric field with an AC voltage amplitude of 3 V over a frequency range of 1–1000 kHz and a measuring electrode with a diameter of 30 mm. Dielectric tests were performed before aging and after each aging cycle to identify the estimated time at which these materials will degrade [45,46].

The temperature resistance was performed to determine the length of time for which these materials can withstand the usual working temperatures. Thus, these materials usually work effectively in a temperature range from −30 °C to 150 °C, which is why we chose to subject the samples to a temperature of 150 °C [47]. This testing was performed using a forced convection oven Memmert model UF 55 (Memmert GmbH + Co. KG., Schwabach, Germany). The disk-shaped samples were subjected to a temperature of 150 °C during five consecutive cycles, each with a duration of 24 h, and the measurements were obtained after cumulative thermal exposures of 48, 72, 96 and 120 h, respectively. After each 24 h cycle, the samples were removed from the oven, placed in a desiccator for cooling and subsequently dielectrically tested. Finally, after 120 h of temperature exposure, the samples were mechanically and structurally tested to identify the changes that occurred.

## 3. Results and Discussion

### 3.1. SEM and EDS Analysis

#### 3.1.1. SEM Analysis

The images resulting from SEM structural analyses of the composites are presented in Figure 3. The magnifications chosen for the analysis were 1000× and 5000×.

The main structural details revealed by SEM images refer to the homogeneity of the dispersion of PZT particles (Figure 3f,h,j). In the case of the composites studied, a homogeneity of the dispersion of PZT powder in the PVDF polymer is observed. Uniform dispersion is essential for obtaining constant electrical properties.

#### 3.1.2. EDS Analysis

**Figure 4:**
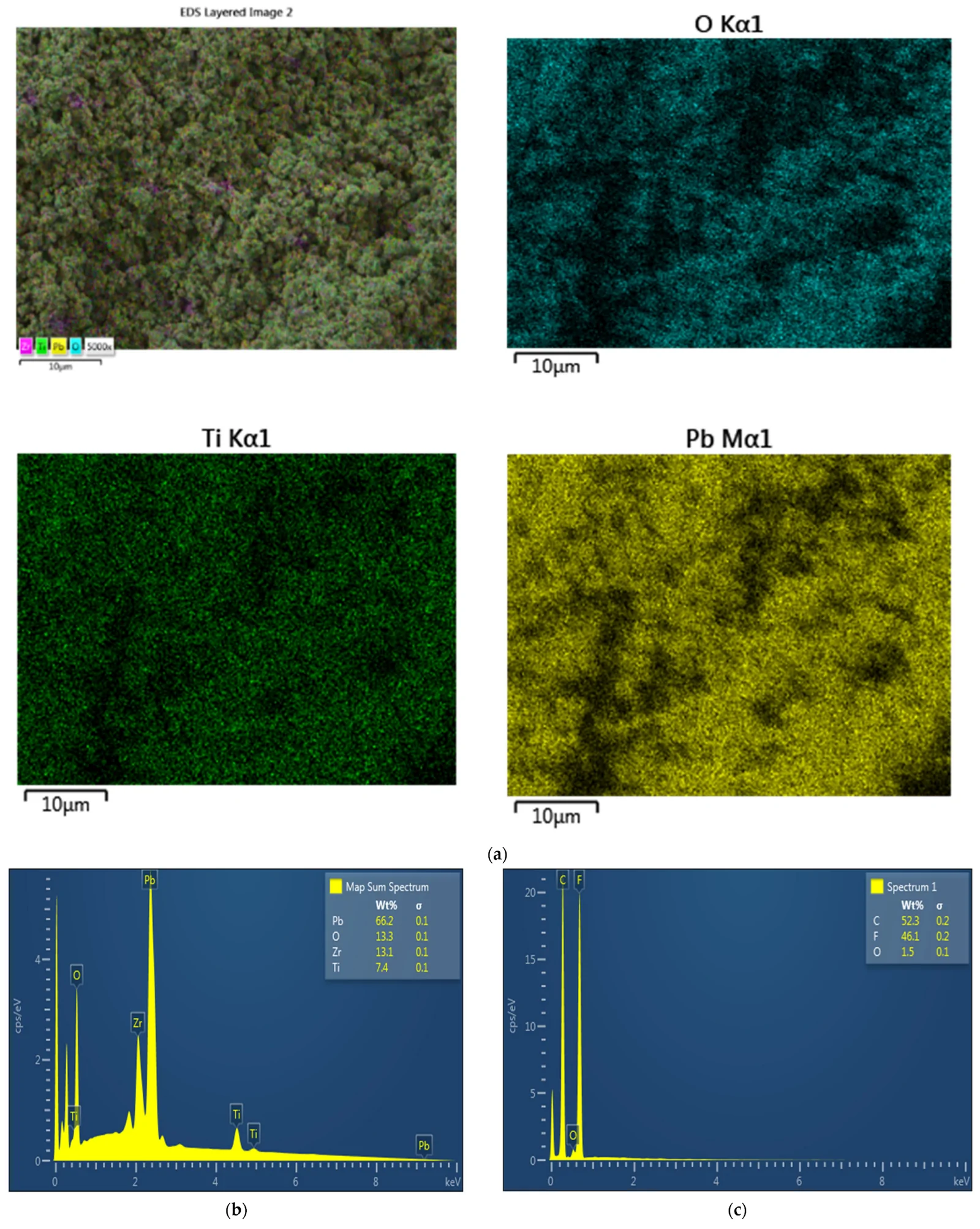

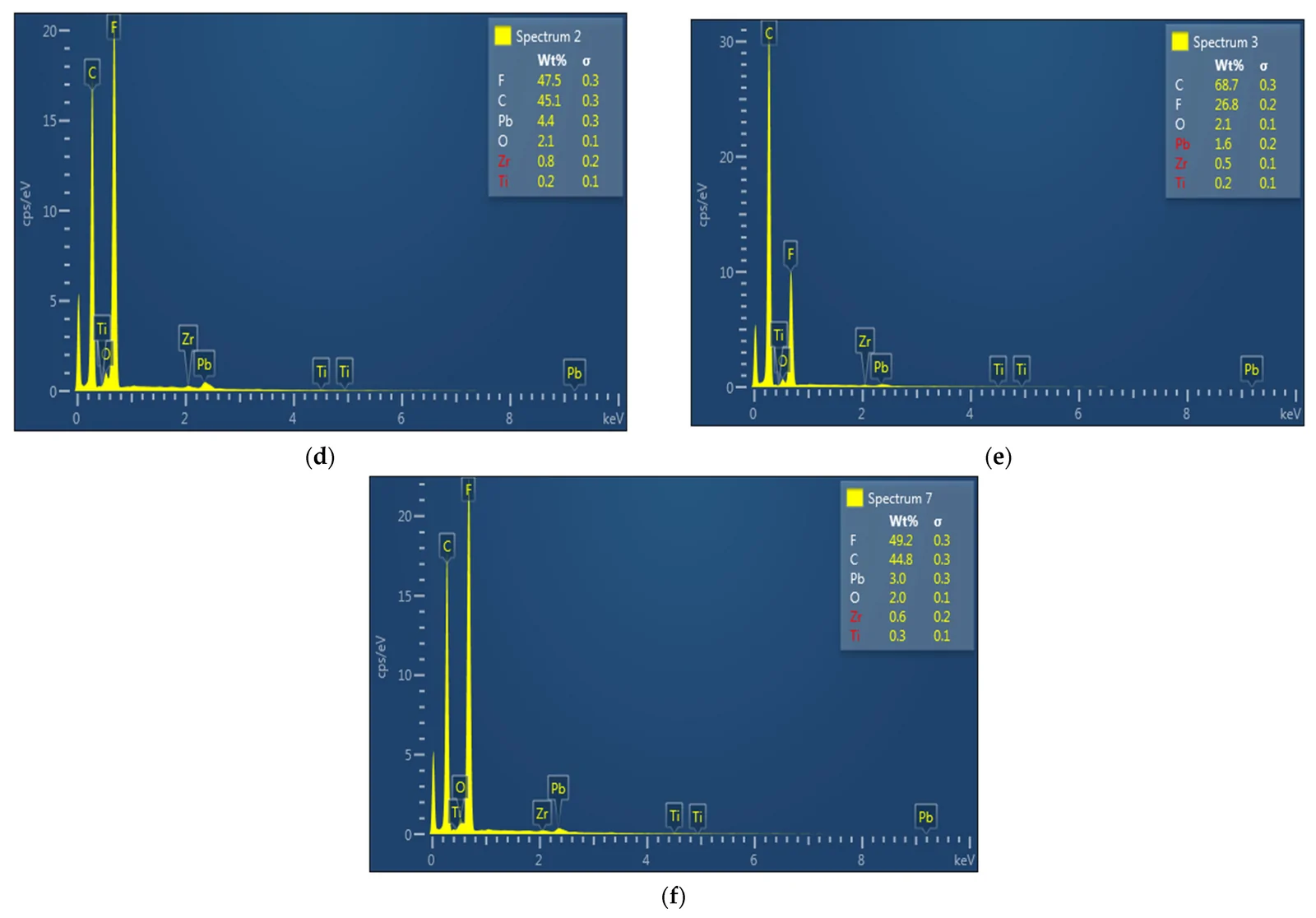
EDS analyses of chemical composition of (**a**) PZT_N2 on constituent elements and areas (**b**) PZT_N2, (**c**) M0, (**d**) M1, (**e**) M2, and (**f**) M3.

This analysis was performed on the surface of the samples. The experimental results obtained from this analysis are presented in Figure 4.

The EDS analysis for these materials aimed to identify the chemical elements (qualitative), which consists of identifying the specific atoms of each phase. Thus, it is observed that for the PZT powder, lead (Pb), zirconium (Zr), titanium (Ti) and oxygen (O) were detected (Figure 4a–c), and for PVDF, fluorine (F) and carbon (C) were detected (Figure 4c–f). The atomic and mass proportion (quantitative) was determined by identifying the exact concentration of each element at the sample surface.

The coupling of SEM with energy-dispersive X-ray spectroscopy (EDX/EDS) provides an elemental chemical mapping. This confirms the presence and distribution of elements in PZT (Pb, Zr, Ti) in relation to light elements in the PVDF matrix (C, F).

### 3.2. FTIR Analysis

**Figure 5:**
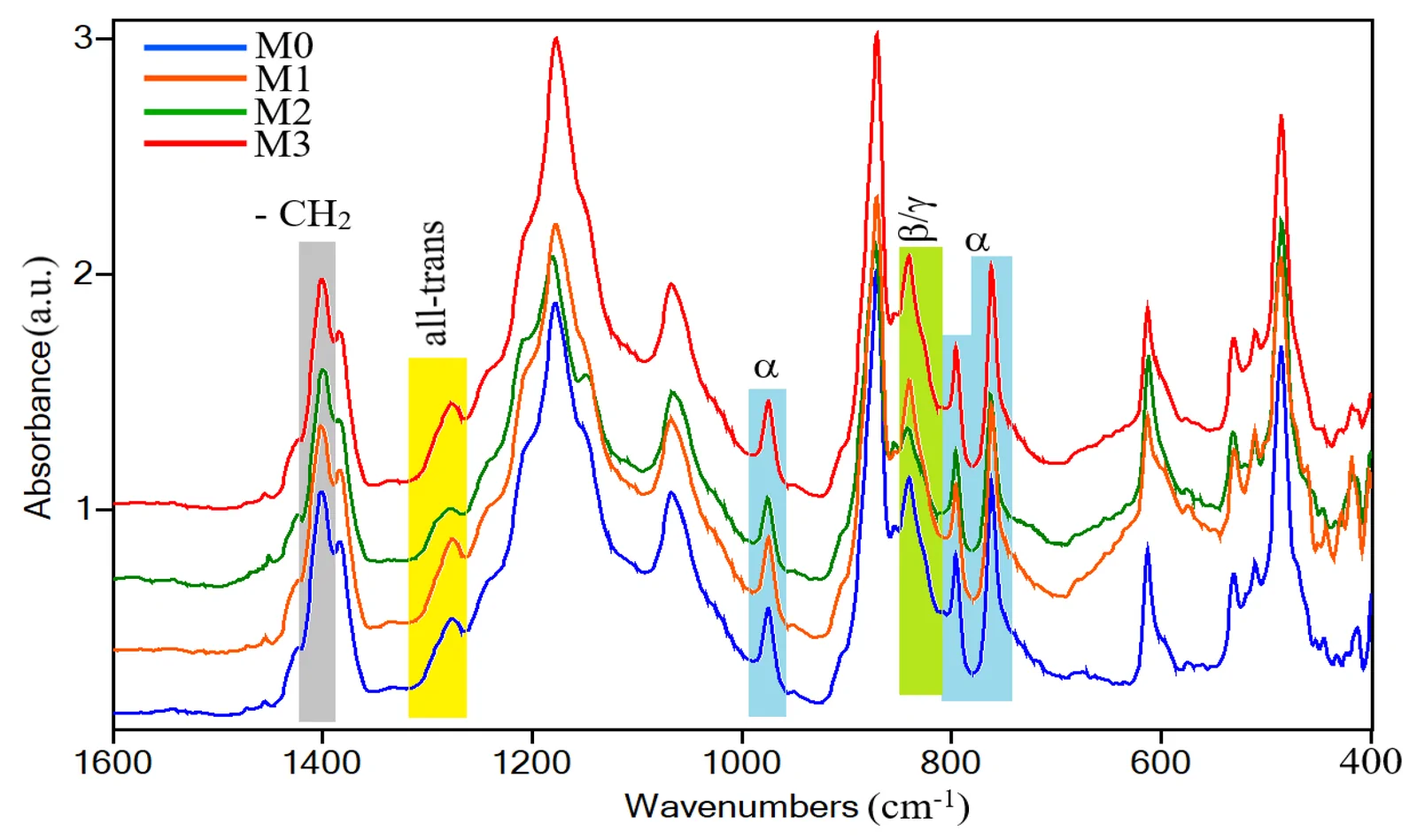
ATR-FTIR spectra of pristine and PVDF/PZT composite samples before thermal aging.

**Figure 6:**
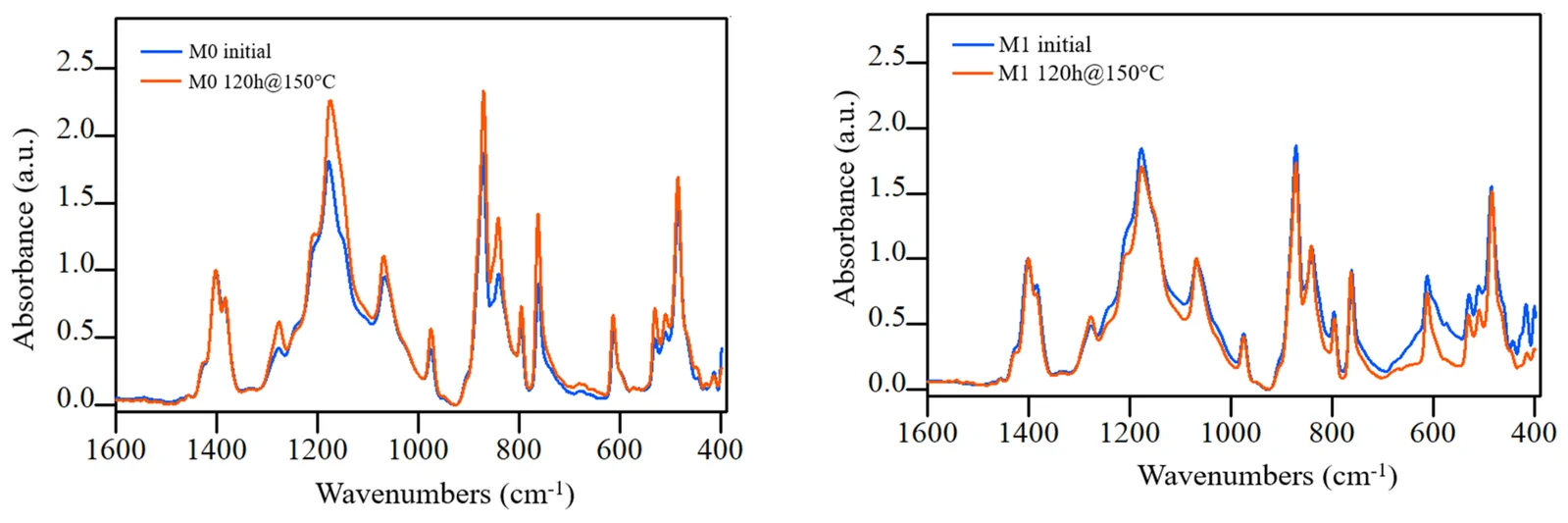

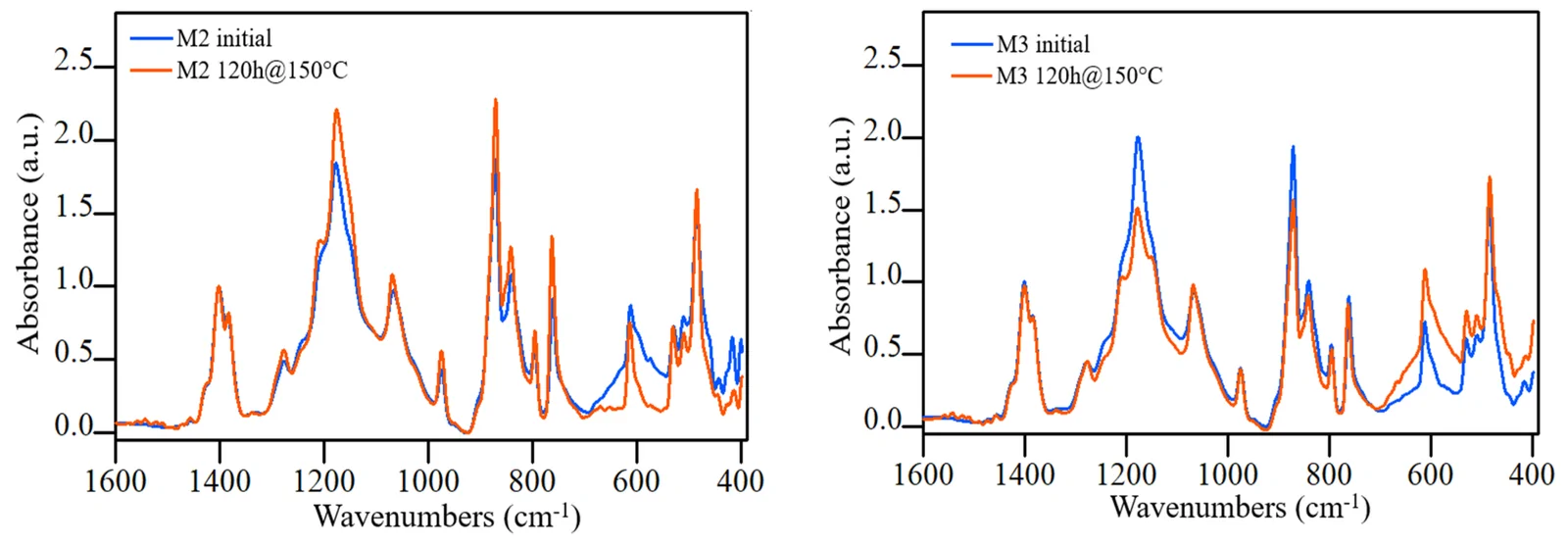
Comparison of the ATR-FTIR spectra before and after thermal aging for each PVDF/PZT composition.

The characteristic PVDF absorption bands (Figure 5 and Figure 6) appear at approximately 485, 530, 612, 763, 795, 840, 875, 975, 1175–1181, 1276–1281 and 1400 cm^−1^ [32,48,49,50]. The bands around 485 and 530 cm^−1^ are commonly linked to CF_2_ bending and rocking motions, together with skeletal vibrations of the PVDF chain. The band near 612 cm^−1^ is mainly associated with CF_2_ bending and chain deformation modes typical of α-PVDF. The peaks at about 763, 795 and 975 cm^−1^ are well-known α-phase markers of PVDF, related to CF_2_ bending/rocking and CH_2_ rocking vibrations. The band around 840 cm^−1^ is generally assigned to CH_2_ rocking coupled with CF_2_ stretching and is widely used as an indicator of electroactive β/γ conformations [51]. The feature near 875 cm^−1^ is usually related to chain vibrations involving CH_2_/CF_2_ rocking and stretching, and it is sensitive to how well the chains are packed and ordered. The band in the 1175–1181 cm^−1^ region is mainly attributed to CF_2_ stretching vibrations and is often discussed in connection with the β/γ spectral region of PVDF. The band at about 1276–1281 cm^−1^ is associated with all-trans chain sequences and is commonly taken as a sign of the electroactive β phase, while the band near 1400 cm^−1^ corresponds to CH_2_ bending and is often used as an internal reference for normalization [50,52].

The incorporation of PZT (Figure 6) did not lead to the appearance of new absorption bands in the organic region and did not induce major systematic shifts in the PVDF bands. This suggests that the interaction between the polymer matrix and the ceramic phase was mainly physical and interfacial, rather than chemical. The PZT particles most likely acted as interfacial sites that could locally influence chain packing and dipole orientation, without producing detectable chemical changes in the PVDF backbone [53].

The *A*_840_/*A*_763_ ratio and the apparent electroactive fraction (*F*_EA,app_) (Equation (1)) indicate a detectable β/γ contribution in all samples. The apparent electroactive fraction was determined with the formula detailed in [52]:

(1) $$F_{\mathrm{EA},\mathrm{app}}\;=\;\frac{A_{840}}{1.3A_{763}\;+\;A_{840}}$$

where *A*_840_ is the local absorbance intensity of the band associated with the electroactive β/γ contribution, while *A*_763_ is the local absorbance intensity of the α-PVDF band. The coefficient 1.3 is the absorption coefficient ratio used for the semi-quantitative estimation of the apparent electroactive fraction.

For the non-aged composites, the F_EA,app_ values vary between 0.227 and 0.339, without a dependence on the PZT concentration. After thermal aging, the apparent FEA values increase to 0.287–0.377, with M1 showing the highest value, suggesting that a low PZT loading may locally favor the formation or stabilization of polar PVDF conformations. In contrast, M2 shows the lowest apparent electroactive contribution, indicating that the effect of PZT is not governed only by its nominal concentration, but also by dispersion, interfacial contact and composite morphology [53,54].

Thermal aging at 150 °C affects mainly the intensity of the PVDF bands, while their positions remain essentially unchanged. For M0 and M2, the increase in the intensity of the bands located in the 700–1500 cm^−1^ region suggests additional structural reorganization and/or crystallization of the PVDF matrix. For M3, the decrease in the absolute band intensities after aging points to a change in surface morphology or in the PVDF/PZT interfacial arrangement, possibly reducing the fraction of PVDF effectively probed by ATR. The increase in the A_1275_/A_763_ ratio after aging indicates a higher relative contribution of all-trans chain segments. However, the persistence of the α-phase bands confirms that thermal aging does not produce a complete transformation toward the β phase.

### 3.3. Mechanical Tests

Compared to conventional polymers, the mechanical properties of polymer composites are strongly influenced by the strength and stiffness of the fillers, which are usually higher than those of the polymer matrices [55,56]. The experimental data for the unaged and aged samples, which were exposed to temperature (150 °C) for 120 h, are presented in Table 4. The variations in mechanical properties of the unaged and aged composite samples relative to M0 are provided in Table 5.

Based on the data presented in Table 4 and Table 5, it was found that all the unaged composite samples (M1–M3) have a higher Vickers hardness (HV) and a higher resistance to plastic deformation or damage (H_IT_) compared to the reference polymer sample (M0).

The composite coded with M1 showed the highest hardness H_IT_ = 0.172 GPa, HV = 15.755 HV and E_IT_ = 2.250 GPa, while the composite with the lowest hardness was M2 with H_IT_ = 0.136 GPa, HV = 5.894 HV and E_IT_ = 2.043 GPa. These results are consistent with the findings reported in the literature [50,51,57].

In this study, the superior mechanical performance of the M1 composite (5 wt% PZT) compared to both the unfilled polymer (M0) and the composites with higher PZT contents (M2 and M3, with 10 wt% and 20 wt%, respectively), can be attributed to the uniform dispersion of PZT particles. At higher concentrations, the PZT particles tend to agglomerate due to van der Waals attractive forces. These agglomerates promote microcrack initiation and compromise mechanical integrity, especially under harsh environmental conditions [55,56]. Similar trends were observed for the thermally aged samples; the highest mechanical properties were obtained for M1TT, with H_IT_ = 0.186 GPa, HV = 17.0611 and E_IT_ = 2.302 GPa.

These results confirm that, at 5 wt% PZT powder, the interfacial adhesion between the filler and the polymer matrix is optimized, increasing stiffness and strength by restricting the mobility of the polymer chain [56,57]. At higher PZT filler concentrations, the reduced effective supersaturation of the powder and the matrix compromises the efficiency of the reinforcement effect [57].

**Figure 7:**
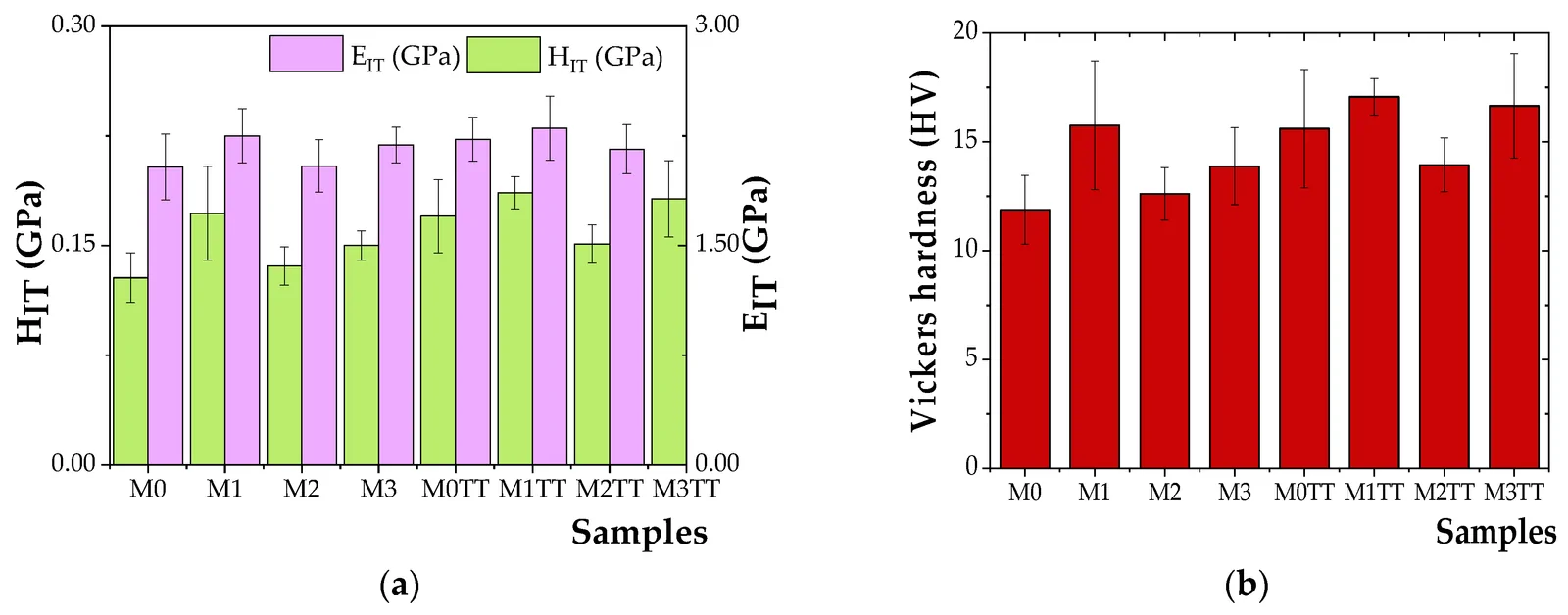
Values of (**a**) indentation hardness (H_IT_), elastic modulus (E_IT_), and (**b**) Vickers hardness HV, and for the unaged and aged samples exposed to a high temperature (150 °C) for 120 h.

Figure 7 shows the comparative variation in the mechanical properties of the materials thermally aged at 150 °C for 120 h compared to those of the initial materials.

### 3.4. Dielectric Tests

#### 3.4.1. Analysis of the Variation with Frequency of Dielectric Parameters tan δ si ε_r_

**Figure 8:**
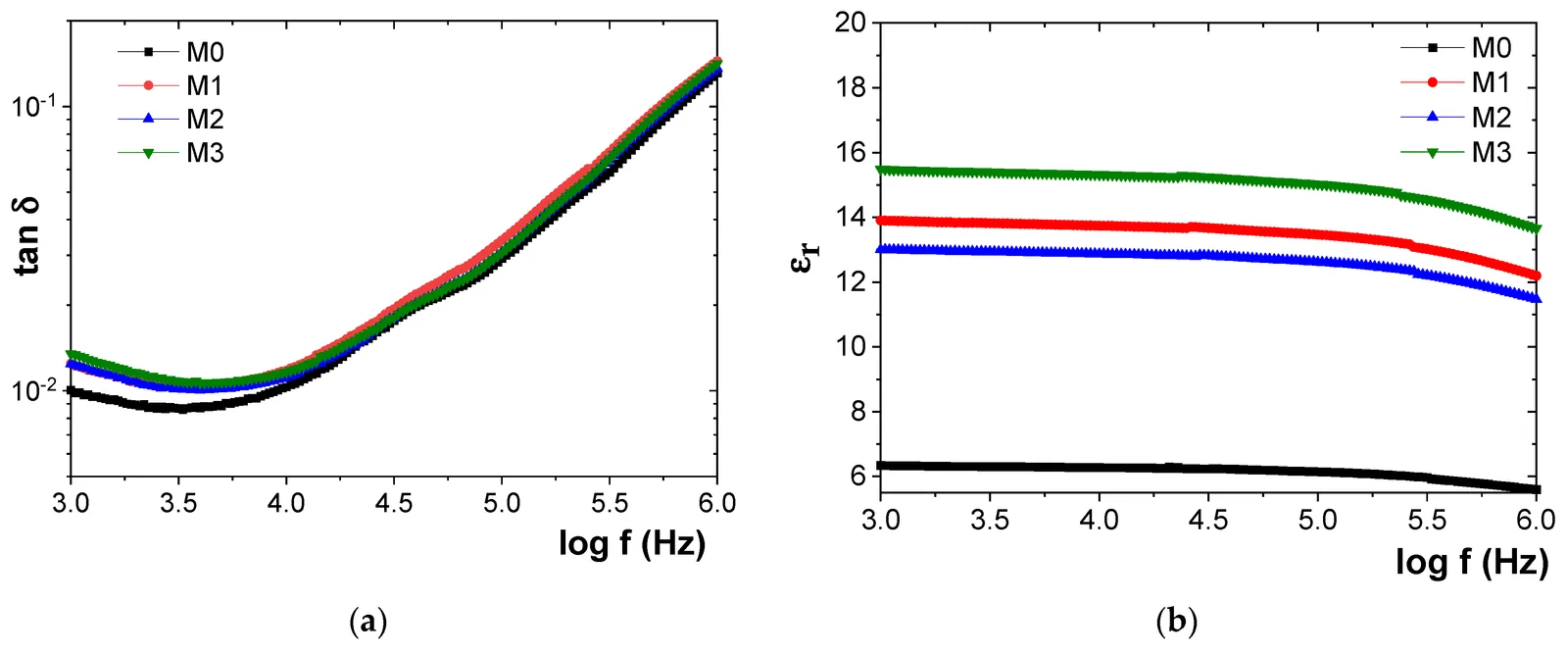
Frequency variation of (**a**) tan δ and (**b**) ε_r_ for materials M0–M3.

The dielectric tests consisted of determining the frequency dependence of the key dielectric characteristics required of an insulator (tan δ, ε_r_, and ρ), followed by the determination of the dielectric breakdown strength (E_str_). Figure 8 shows the initial values of tan δ and ε_r_ as a function of frequency. The values obtained for tan δ and εr at 1, 10, 100, and 1000 kHz are presented in Table 6.

In the case of the composite material designated M2, the addition of PZT to the polymer composition led to an increase in dielectric losses and lattice defects, which implicitly resulted in higher electrical conductivity. In contrast, materials M1 and M3 exhibited the lowest dielectric losses (among the three analyzed composite materials—M1, M2, and M3); therefore, it can be stated that, although a 5% PZT proportion is relatively low, the interface between the ceramic phase (PZT) and the polymer matrix (PVDF) radically altered the electrical behavior [53,54,57]. It was observed that very similar curves were obtained (for tan δ), which can be explained by the predominant polymer content.

Table 6 shows that for PZT/PVDF composites, the relative permittivity (ε_r_)—measured at room temperature and a frequency of 1 kHz—increases steadily by approximately 59% as the PZT concentration rises. A significant increase in tan δ values of approximately 25% is also observed. The dielectric behavior is primarily influenced by the polymer matrix, as interfacial charge accumulation is minimal.

#### 3.4.2. Analysis of the Frequency Dependence of the Dielectric Parameters tan δ and ε_r_ for Samples Subjected to Temperature

**Figure 9:**
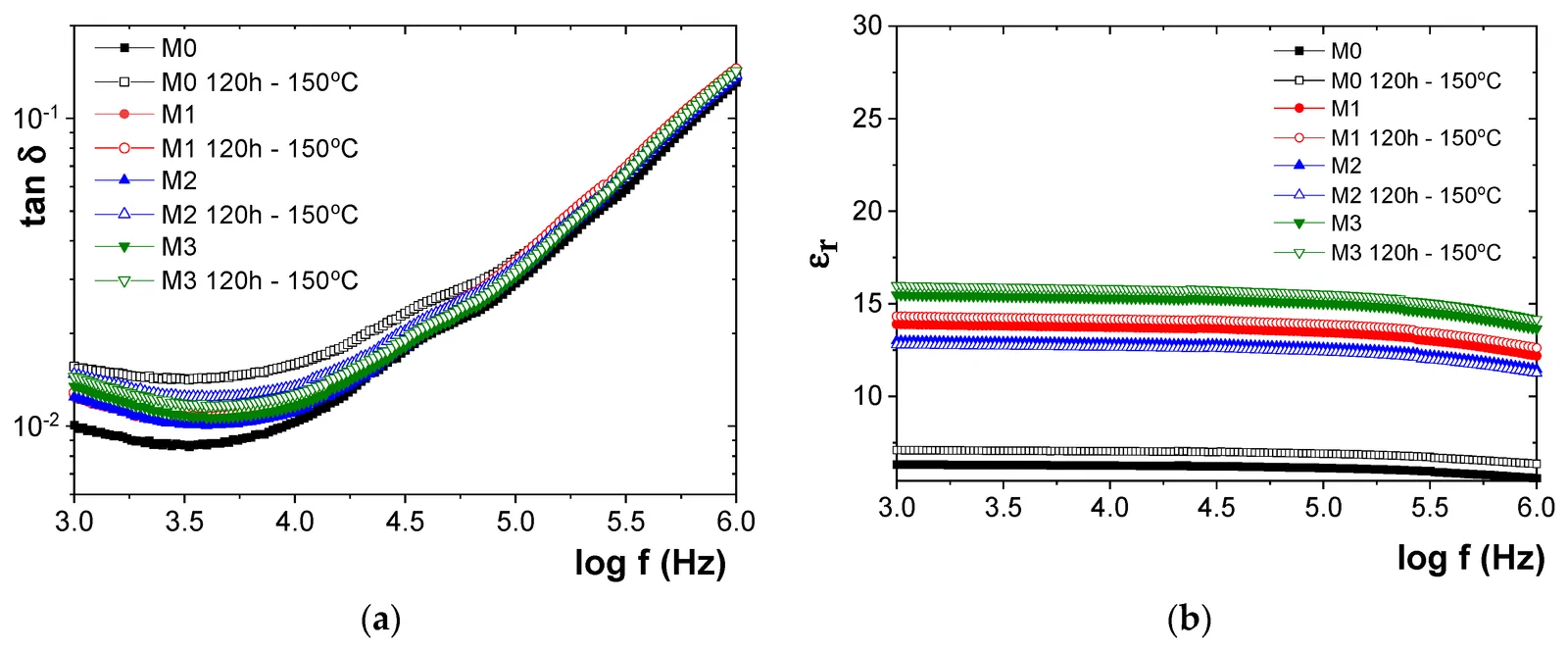
Comparative presentation of the variation as a function of frequency of (**a**) tan δ and (**b**) ε_r_ initially and for 120 h aging at a temperature of 150 °C.

The experimental values of tan δ and ε_r_ were presented comparatively (Figure 9), the initial values and those after 120 h.

Incorporating PZT into the PVDF matrix resulted in a gradual and moderate decrease in volume resistivity compared to pure PVDF (M0). This change was attributed to interfacial polarization and the characteristics of the ceramic phase. The resistivity at 1000 kHz of the composite material with the maximum PZT concentration (M4) decreased by approximately 13% compared to the PVDF polymer matrix (M0).

##### Determining the Remaining Service Life

**Figure 10:**
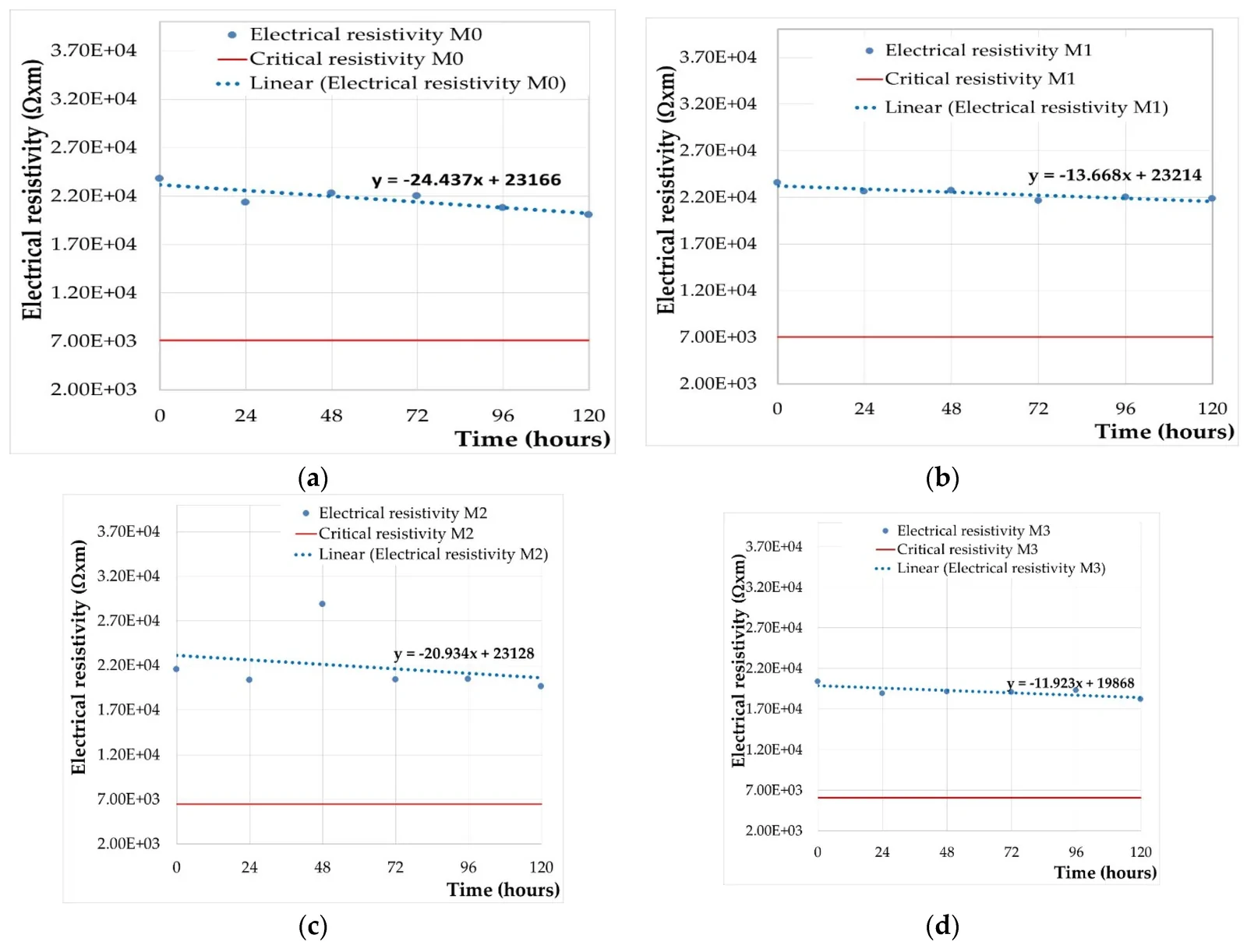
Trends in electrical resistivity variation for M0–M3.

The remaining lifetime is very important for the equipment insulated with the composite materials described in this work, because it estimates the time that remains until the complete degradation of the material occurs. Upon reaching the end of its lifetime, the material should be replaced to ensure that it does not affect the operation of the equipment; as a worst-case scenario, failing to replace it could lead to damage. It is observed that if the conductivity increases with the addition of PZT powder, the resistivity decreases in the same direction. The experimental results of the tests conducted to determine these materials’ resistance to temperature were obtained, and the remaining lifetime until degradation was identified for each one. The variation in the electrical resistivity of the composites at a frequency of 1 MHz during different periods of exposure to temperature is presented in Figure 10.

**Figure 11:**
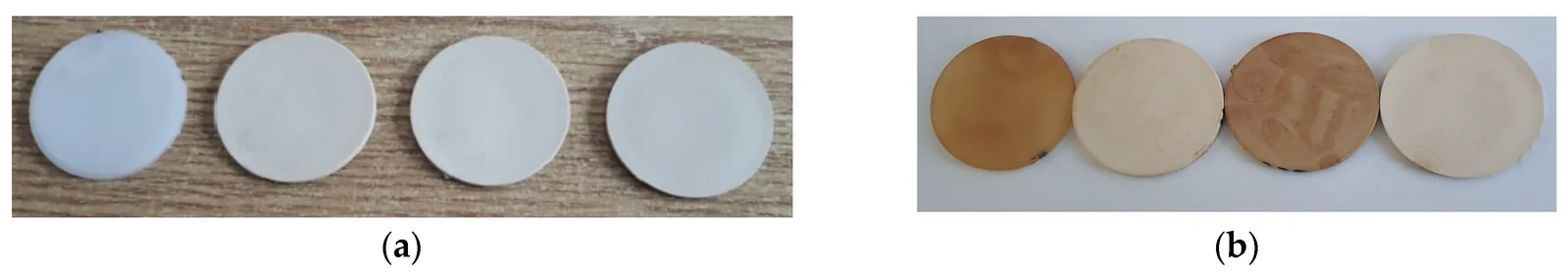
Materials studied (from left to right) M0, M1, M2 and M3. (**a**) Initial state; (**b**) aged 120 h at 150 °C.

The critical resistivity was calculated as 30% of the electrical resistivity value at 1000 kHz. The first-degree equations were extracted from the graphical representations, which describe the trend of the variation curve (Figure 11) from which the data in Table 7 are obtained. The remaining operating life under normal conditions was calculated by subtracting the 120 h of temperature exposure from the number of hours obtained for each material. Based on these times, the composite that degrades the fastest could be identified.

The values in the “Remaining lifetime until degradation” column were calculated by subtracting the 120 h for which the samples were aged from the values in the “lifetime” column. The nature polymer M0 was the least resistant to temperature action. Analyzing the behavior of the composites (M1–M3), it was found, from Table 8, that a classification of the composites could be made according to the temperature resistance as follows: RM1 > RM3 > RM2. This is evidenced by the remaining lifetime until the total degradation of the material. For M1 it was 1065 h, for M2 it was 676 h, and for M3 it was 1033 h. This behavior is due to the fact that in the process of obtaining composite materials from the melt in the polymer mixture and the PZT filler powder, various mechanical interactions took place due to the strong polarization of the filler, which caused slight repulsion forces. As a result, micron-sized spaces were created between these particles, which were partially occupied by the PZT particles. Thus, following the experiments carried out, a comparison could be made between the remaining lifetime of the composites and the control material made of PVDF (M0). Thus, it was found that for M1, the lifetime increased by approx. 44%; for M2 the lifetime increased by approx. 17%; and for M3 the lifetime increased by approx. 43%. From this analysis, it was found that adding percentages higher than 5% of PZT powder did not lead to an increase in the lifetime in response to temperature exposure; on the contrary, a decrease in the lifetime was found. So it can be said that the optimal percentage at which an increase in resistance to the action of temperature was obtained was 5%, respectively, as this was the percentage of PZT powder used to obtain the composite coded with M1.

In Figure 11, the initial materials are presented in comparison with those subjected to a temperature of 150 °C for 120 h.

### 3.5. Determination of Electrical Resistivity

The electrical resistivity was determined by a calculation based on the experimental results obtained from the dielectric tests performed on these materials. The tangent of dielectric loss angle (tan δ) or dissipation factor was determined as the ratio between the imaginary part (ε_r_″) and the real part of the relative permittivity (ε_r_′) as shown in Equation (2):

tan δ = ε_r_″/ε_r_′ (2)

The real parts of electrical conductivity (σ, in S/m) and resistivity (ρ, in Ω⋅m) were calculated with Equations (3) and (4):

σ = ω ε_0_ ε_r_ tan δ (3)

ρ = 1/σ(4)

where

ε_0_ is the permittivity of free space (8.854 × 10^−12^ F/m);
ε_r_′ is the relative permittivity (dielectric constant) of the sample;
d is the thickness of the sample (m);
ω is the angular frequency (ω = 2πf);
f is the linear frequency;
tan δ is the tangent of dielectric loss angle (dissipation factor).

The electrical conductivity was calculated according to formula 3 and the electrical resistivity was calculated as the inverse of the electrical conductivity according to relation 4.

Thus, in Table 9, the electrical resistivities at a frequency of 1 kHz between the initial materials and those aged for 120 h at 150 °C are compared.

Due to the fact that the highest concentration of these materials is that of the PVDF polymer, the values are quite close. From Table 9 it can be seen that, in the case of unaged materials, the highest electrical resistivity (ρ) is M1, and the same trend is observed for thermally aged composites. That is, the highest electrical resistivity among the unaged materials is shown by M1, whereas among the aged ones it is shown by M1 120 h–150 °C. So it can be concluded that among the analyzed composite materials, the composite material M1 (PVDF/5%PZT) has the highest resistivity, so from the perspective of the chosen application (as cable insulation), the material M1 is the optimal variant among the analyzed composites.

### 3.6. Determination of Dielectric Strength

The dielectric breakdown strength of the PZT/PVDF composites reduces as the PZT content rises up to 20%. This reduction is mainly attributed to the significant mismatch in dielectric constants between the ceramic filler and the polymer matrix. However, the decrease reaches a maximum of only 30%, as the volume fraction stays low and the polymer matrix continues to maintain a continuous high-breakdown network.

Table 10 presents the breakdown voltage values for the analyzed materials.

When comparing the experimental data with the specified requirements for applications in electrical insulation, the results show that all materials meet the criteria. Specifically:

1. They exhibit a high dielectric permittivity greater than 10.
2. They possess a high dielectric breakdown strength, exceeding 190 kV/mm (with a minimum requirement of 100 kV/mm).
3. They demonstrate low dielectric loss below 0.0235 (where the minimum acceptable is less than 0.05).
4. They have high volume resistivity and a low leakage current, registered in Ohm m values.
5. They show interfacial compatibility and homogeneous dispersion, as proven by imaging analysis.
6. They maintain thermal stability with a minimum of 130 °C while ensuring optimal dielectric performance.

## 4. Conclusions

In this study, four materials—three composites and the base polymer PVDF—were produced via melt injection and subsequently characterized.

The mass concentrations, expressed as the PVDF/PZT ratio, were 100/0, 95/5, 90/10, and 80/20. The materials underwent structural, thermal, mechanical, and dielectric characterization to identify the optimal composite variant for specialized insulation applications. High-temperature resistance was demonstrated through thermal endurance tests at 150 °C using 24 h cycles, determining the remaining service life prior to material degradation.

The characterization results showed the following: SEM analysis indicated a relatively uniform distribution of PZT powder within the PVDF polymer matrix; EDS analysis revealed surface concentrations of the ceramic phase of approximately 5% for M1, 2% for M2, and 4% for M3; and mechanical testing identified the M1 composite (PVDF + 5% PZT) as the optimal variant.

To demonstrate the suitability of the composites as insulating materials, specific dielectric tests (tan δ, ε_r_, ρ, and E_str_) were performed. All characterization results confirmed that the composite material containing 5% PZT powder represents the optimal choice for insulation applications.

Primary Targeted Applications:

1. Electric Field Grading and Stress Control Coatings: These are used in high-voltage cable terminations, bushings, and switchgear to even out electric field distributions, reduce localized electric stress peaks, and prevent issues like partial discharge or surface flashovers.
2. High-Voltage Transient and Pulse Insulation: Integrated into specialized pulsed-power systems and high-frequency transformer insulation layers, requiring high spatial capacitance combined with strong voltage tolerance. The required properties for dielectric insulation are as follows:
  − High Dielectric Permittivity: PZT ceramic fillers are used to significantly enhance the composite permittivity beyond that of PVDF, enabling a high capacity for charge storage.
  − High Dielectric Breakdown Strength: The material must endure intense electric field stress without failing electrically. Maintaining low field concentrations at filler–matrix interfaces helps achieve high breakdown levels of around 100 kV/mm.
  − Low Dielectric Loss: This reduces internal heat generation and avoids thermal runaway during high-frequency or high-voltage AC operations, aiming for a tan delta less than 0.05.
  − High Volume Resistivity and Low Leakage Current: A high electrical insulation resistance (10^12^) is essential to prevent DC leakage current and minimize active power dissipation.
  − Interfacial Compatibility and Homogeneous Dispersion: PZT particles should be evenly distributed within the PVDF matrix, avoiding agglomeration or micro-voids that could lead to localized charge accumulation.
  − Thermal Stability: The material must retain high thermal stability up to its operational limits (typically between 120 and 150 °C) while maintaining optimal dielectric performance.

## Figures and Tables

**Table 1:** Electrical properties for the PZT doped with Nb^5+^ and Sr^2+^ and sintered at 1140 °C for 2 h measured at room temperature (22 ± 3 °C).

Relative Dielectric Permittivity (ε_r_ = ε^T^_33_/ε_0_)	Dielectric Loss Factor (tanδ) [×10^−3^]	Electromechanical Coupling Factor (k_p_)	Direct Charge Coefficient (d_33_) [×10^−12^ mV^−1^]
1180	14	0.42	180

**Table 2:** Concentrations and codings.

Concentration (% wt)/Coding	M0	M1	M2	M3
PVDF	100	95	90	80
PZT	0	5	10	20

**Table 3:** Temperature regime for obtaining materials.

Zone	5	4	3	2	1
Temperature (°C)	250	240	230	220	210

**Table 4:** Mean ± SD values of indentation hardness (H_IT_), Vickers hardness HV, and elastic modulus (E_IT_) for the unaged and aged samples exposed to humidity and high temperature (150 °C) for 120 h.

Samples	H_IT_ (GPa)	Errors H_IT_ (GPa)	E_IT_ (GPa)	Errors E_IT_	Vickers Hardness HV	Errors HV	C_IT_ (%)	Errors C_IT_ (%)	η_IT_(%)	Erori η_IT_(%)
M0	0.128	±0.017	2.037	±0.225	11.873	±1.572	7.084	±2.088	33.601	±4.136
M1	0.172	±0.032	2.250	±0.184	15.755	±2.958	6.524	±0.360	43.299	±1.745
M2	0.136	±0.013	2.043	±0.179	12.613	±1.207	5.894	±0.389	40.914	±2.782
M3	0.15	±0.010	2.187	±0.121	13.878	±1.768	6.43	±0.214	42.318	±0.800
M0TT	0.17	±0.025	2.226	±0.151	15.604	±2.720	3.504	±0.761	44.367	±1.527
M1TT	0.186	±0.011	2.302	±0.220	17.061	±0.834	6.375	±1.250	40.856	±1.027
M2TT	0.151	±0.013	2.158	±0.168	13.939	±1.242	6.512	±0.385	45.472	±1.137
M3TT	0.182	±0.026	2.219	±0.156	16.651	±2.404	3.19	±0.045	47.673	±1.169

**Table 5:** Variation in mean values of indentation hardness (H_IT_), Vickers hardness HV, and elastic modulus (E_IT_) of the unaged and aged composite samples relative to M0.

Mechanical Property	Increase from M0 (%)						
	M1	M2	M3	M0 TT	M1 TT	M2 TT	M3 TT
H_IT_ (GPa)	25.58	5.88	14.67	24.71	31.18	15.23	29.67
E_IT_ (GPa)	9.47	0.29	6.86	8.49	11.51	5.61	8.20
Vickers hardness HV	24.64	5.87	14.45	23.91	30.41	14.82	28.69

**Table 6:** Tan δ/ε_r_ values at frequencies of 1000, 10,000, 100,000 and 1,000,000 Hz.

Frequency (kHz)/Material	1		10		100		1000	
	tan δ	ε_r_	tan δ	ε_r_	tan δ	ε_r_	tan δ	ε_r_
M0	0.0101	6.3391	0.0103	6.2788	0.0293	6.1482	0.1313	5.5970
M1	0.0125	13.9011	0.0118	13.7377	0.0338	13.4598	0.1445	12.1919
M2	0.0124	12.9995	0.0111	12.8664	0.0308	12.6147	0.1384	11.4683
M3	0.0135	15.4816	0.0116	15.3175	0.0306	15.0231	0.1415	13.6515

**Table 7:** Electrical resistivity (**ρ**) at 1000 kHz.

Hours	ρ M0	ρ_cr_ M0	ρ M1	ρ_cr_ M1	ρ M2	ρ_cr_ M2	ρ M3	ρ_cr_ M3
0	23,428.30	7028.49	23,400.78	7020.23	21,542.73	6462.82	20,385.27	6115.58
24	21,066.74	7028.49	22,484.28	7020.23	20,356.01	6462.82	18,927.96	6115.58
48	21,989.75	7028.49	22,585.41	7020.23	28,836.32	6462.82	19,092.68	6115.58
72	21,983.10	7028.49	21,528.24	7020.23	20,373.73	6462.82	19,049.33	6115.58
96	20,759.59	7028.49	21,874.98	7020.23	20,477.03	6462.82	19,280.72	6115.58
120	20,068.57	7028.49	21,708.71	7020.23	19,645.67	6462.82	18,179.29	6115.58

**Table 8:** Evaluation of the lifetime until degradation.

Samples	Equation	Intercept = y	a	b	Lifetime (h)	Remaining Lifetime Until Degradation (h)
M0	y = −24.437x + 23,166	7.03 × 10^3^	−24.437	2.32 × 10^4^	660	540
M1	y = −13.668x + 23,214	7.02 × 10^3^	−13.668	2.32 × 10^4^	1185	1065
M2	y = −20.934x + 23,128	6.46 × 10^3^	−20.934	2.31 × 10^4^	796	676
M3	y = −11.923x + 19,868	6.12 × 10^3^	−11.923	1.99 × 10^4^	1153	1033

**Table 9:** Variation in electrical resistivity.

Hours/ρ	0 h	120 h
M0	2.41 × 10^8^	1.66 × 10^8^
M1	2.58 × 10^8^	2.33 × 10^8^
M2	1.94 × 10^8^	1.13 × 10^9^
M3	2.31 × 10^8^	1.88 × 10^8^

**Table 10:** Variation in dielectric strength.

Samples	Estr (kV/mm)
M0	270
M1	250
M2	225
M3	190

## Data Availability

The data are presented in this study.
